# The Association of Dietary Diversity with Hyperuricemia among Community Inhabitants in Shanghai, China: A Prospective Research

**DOI:** 10.3390/nu16172968

**Published:** 2024-09-03

**Authors:** Xiaoli Xu, Mengru He, Genming Zhao, Xing Liu, Xiaohua Liu, Huilin Xu, Yuping Cheng, Yonggen Jiang, Qian Peng, Jianhua Shi, Dandan He

**Affiliations:** 1 Minhang District Center for Disease Control and Prevention (Minhang District Institute of Health Supervision), Shanghai 201101, China; lisa861227@126.com (X.X.);; 2 School of Public Health, Fudan University, Shanghai 200032, China; 3 Songjiang District Center for Disease Control and Prevention, Shanghai 201600, China; 4 Jiading District Center for Disease Control and Prevention, Shanghai 201800, China; 5 Xuhui District Center for Disease Control and Prevention, Shanghai 200237, China

**Keywords:** hyperuricemia, dietary diversity, prospective cohort study

## Abstract

Hyperuricemia, a major worldwide burden on public hygiene, is closely connected with dietary habits. However, few studies have evaluated the association of dietary diversity with hyperuricemia. To preliminarily reveal the status of a diversified diet in preventing hyperuricemia based on a neighborhood-based, massive-scale cohort in China, a total of 43,493 participants aged 20–74 years old, with no history of hyperuricemia at baseline, were enrolled in the research from April 2016 to December 2019. The Dietary Diversity Score (DDS) was utilized to evaluate the dietary variety and split the participants into the low-, medium-, and high-DDS groups. Information on participants was connected to regional health information systems that acquired data on hyperuricemia instances up to 28 February 2023. Hazard ratios (HRs) and 95% confidence intervals (CIs) were computed by Cox proportional hazards models. Restricted cubic splines (RCS) were implemented to analyze dose–response correlation. A total of 1460 individuals with newly diagnosed hyperuricemia were observed over a median follow-up period of 5.59 years. Compared to the low-DDS group, HRs for the medium- and high-DDS groups were 0.87 (95% CI 0.76–0.99) and 0.80 (95% CI 0.70–0.91) in the fully adjusted model, respectively. The risk of hyperuricemia incidence was reduced by 5% for each 1 unit of DDS increase. A linear correlation of DDS with hyperuricemia emerged and further revealed that the intake of 8–10 broad categories of food could decrease the incidence of hyperuricemia. Our results validate the dietary principle of “food diversification” recommended in guidelines. Conclusions should be applied with caution considering the paucity of related evidence in additional nations.

## 1. Introduction

Hyperuricemia, represented by the presence of increased uric acid levels, has been identified as a significant contributor to the risk for multifarious chronic diseases, including gout [1], type 2 diabetes mellitus [2], heart vessel disease [3], and renal disease [4]. Moreover, a rising amount of evidence has established a connection between hyperuricemia and all-cause mortality or even premature demise [5,6,7]. The prevalence rate of hyperuricemia remained high at almost 20% from 2007 to 2016 in the United States [8]. Similar rates of hyperuricemia were disclosed in Ireland and Australia [9,10]. A recent nationwide study in China disclosed a total hyperuricemia prevalence of 14.0% of Chinese adults (≥18 years old), which appears to be an alarming uptrend compared to the hyperuricemia prevalence of 8.4% ten years ago [11]. Hyperuricemia, as a significant public health concern in the world, has turned into the “fourth highest” burden after hypertension, diabetes, and hyperlipidemia, and the disease burden is also expected to increase [6,12].

A long-term increase in hyperuricemia is attributed to multiple factors, especially dietary habits. Studies have revealed that consumption of some dietary elements in high-purine diets such as red meat and seafoods corresponded to an elevated risk of hyperuricemia [12,13,14]. Similarly, a healthy habit like consuming more vegetables and fruits was also correlated with a decreased risk of hyperuricemia [15]. However, overemphasizing the restricted consumption of certain sustenance or nutrients could cause an imbalance in nutrients by substituting unwholesome carbon-refined carbohydrates for protein [16]. More recently, studies revealed that certain dietary protocols, consisting of the Mediterranean diets and dietary approaches to stop hypertension (DASH), may decrease concentrations of serum uric acid and prevent conditions of hyperuricemia [13,17]. A varied and balanced diet should be chosen to improve general health and prevent hyperuricemia rather than depending exclusively on a specific dietary pattern [13].

Dietary diversity is a crucial aspect of diet with exceptional quality generally encouraged and recommended within the framework of dietary advice and dietary guidelines in different nations around the world [18,19,20,21,22]. The Dietary Diversity Score (DDS) is considered as a commonly employed worldwide indicator [23] that assesses the entire diet to represent the food quality and nutritional adequacy [24,25]. Numerous studies confirmed the correlation of DDS with multiple types of health consequences, including chronic diseases (such as diabetes and heart disease), cognitive impairment and vulnerability, and all-cause mortality [26,27,28,29,30]. However, as far as we are aware, few studies have reported on the correlation between dietary diversity and hyperuricemia. To resolve this research disparity and preliminarily expose the preventive impact of a diversified diet on hyperuricemia, we implemented this analysis depending upon the Shanghai Suburban Adult Cohort and Biobank (SSACB) database.

## 2. Materials and Methods

### 2.1. Study Design and Population

Prospective and community-based cohort data from the SSACB were utilized. A detailed description of the SSACB can be found in a prior publication [31]. In simple terms, twelve communities in each of Shanghai’s four districts (Jiading, Minhang, Songjiang, and Xuhui) were initially selected pursuant to population density and financial condition. Afterwards, approximately one in three of the villages and committees were chosen at random within each district as research sites. Inhabitants who were in the age range from 20 to 74 years old and had resided in Shanghai for more than 5 years were recruited by clustered random sampling method as participants.

In total, 69,116 individuals participated in the baseline phase of this research between April 2016 and December 2019. All participants completed a structured questionnaire survey and underwent a standardized physical examination conducted by trained professional technicians and professional clinicians, respectively. Demographics (consisting of gender, age, marital situation, education attainment, and retirement status), medical histories, lifestyle information [consisting of smoking status, alcohol consumption, diet, tea intake, physical activity (PA), and sleep], and anthropometric measurements were collected. Each individual additionally had corresponding biochemical analyses performed on their fasting blood and urine samples, both of which were preserved in a −80 °C refrigerator for no longer than 6 h until they were delivered to the national diagnostic company [Dian Diagnostics Co., Ltd. (Hangzhou, China)] for further analysis. Each individual with a unique identification code was linked to the Electronic Medical Record System (EMR), the Chronic Disease Management System (CDM), the Cancer Registry System (CR), and the Cause-of-Death Surveillance System (CDSS), where the coding standard of the International Classification of Diseases tenth revision (ICD-10) is implemented for disease diagnosis [17,31].

**Figure 1:**
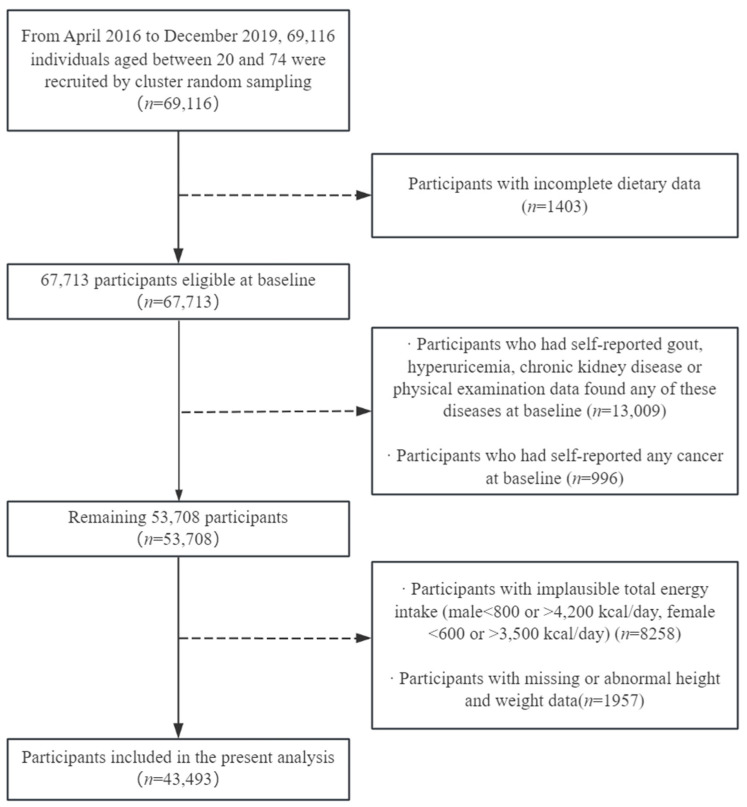
Flow diagram of recruitment steps for participants.

After the elimination of 1403 participants with insufficient reported dietary information, 13,009 participants with self-reported gout, hyperuricemia, or chronic kidney disease, or physical examination data that found any of these diseases at baseline, 996 participants with self-reported cancer of any type, 8258 subjects with ridiculous overall energy intake (for males < 800 or >4200 kcal/day, for females < 600 or >3500 kcal/day) [32], and 1957 subjects with missing or abnormal height and weight, a total of 43,493 Shanghai-based individuals (15,727 male and 27,766 female) aged between 20 and 74 years were embraced within the scope of the present research (Figure 1). The research procedure was authorized by the Ethical Review Committee of the School of Public Health, Fudan University (IRB approval number 2016-04-0586). All subjects provided written informed approval prior to their participation.

### 2.2. Evaluation of Dietary Diversity

Considering Shanghai’s customary eating patterns, a semi-quantitative food frequency questionnaire (FFQ) consisting of 29 categories of foods was utilized to analyze the dietary consumption based on participants’ responses recalling the frequency and average consumption of food in the preceding 12 months [17]. Eight gradations of dietary frequency were delineated: “never”, “fewer than once per month”, “1–3 times per month”, “1–3 or 4–6 times per week”, and “once, twice, or more than thrice daily”.

DDS served as a metric for gauging the variety within the dietary intake of the participants, which was established in accordance with a previous study [33,34], the authoritative Dietary Guidelines for Chinese Residents (2022) [35], and the Food and Agriculture Organization of the United Nations Guidelines for Grain Classification [23]. All foods were classified into cereals, tubers, fruits, vegetables, fish, meat, soybeans and their products, milk and dairy products, eggs, mushrooms, nuts, salt, and oils. Considering that cereals, oils, and salt are consumed almost every day, the evaluation of DDS in this study was calculated on the consumption frequency of the remaining 10 kinds of foods [29,36]. One DDS unit was identified as “1–3 or 4–6 times per week” or “once, twice, or more than thrice daily” intake of any food group without taking into account a minimum consumption. The highest attainable DDS was 10 points, referring to the greatest degree of dietary diversity. Furthermore, for each participant, the DDS based on tertiles was divided into the low-, medium-, and high-DDS groups.

### 2.3. Follow-Up and Ascertainment of Hyperuricemia

Follow-up was conducted depending on the connectivity to health information systems such as the EMR and CDM that meticulously document the identities and dates of disease diagnoses. Hyperuricemia was recognized as elevated serum uric acid ≥ 420 μmol/L in males and ≥360 μmol/L in females [11,37] or when diagnosed by licensed physicians as hyperuricemia (E79) in the systems.

### 2.4. Assessment of Covariates

Sociological demographic characteristics, lifestyle information, and physiological status information served as covariates in our study. The adjusted sociological demographic characteristics covered age (years old), gender (male or female), education attainment (“primary school and below”, “junior high school”, or “senior high school and above”), marital situation [“unmarried”, “married”, “other circumstances (including divorce, widowhood, etc.)”], and retirement status (yes or no). The definition of retirement was based on whether or not the person had retired at the time of investigation. Lifestyle information comprised smoking status (yes or no), alcohol consumption (yes or no), tea intake (yes or no), dietary energy intake, PA (low, medium, or high level), and sleeping time (<5 h, 5–8 h, or ≥8 h). Smoking extent was established by the answer to the query “Have you ever consumed cigarettes at a minimum per day for at least six uninterrupted months?” Alcohol consumption extent was established by the answer to the query “Have you ever consumed alcohol no less than 3 times per week for at least six uninterrupted months?” Tea intake status was established by the answer to the query “Have you ever consumed tea no less than 3 times per week for at least six uninterrupted months?” Dietary energy intake was calculated based on the quantitative consumption of 29 categories of foods and the Chinese Food Composition Table [38]. PA was evaluated based on the short International Physical Activity Questionnaire (IPAQ) form and categorized into three classifications: “low level”, “medium level”, and “high level” [39].

Physiological status information covered body mass index (BMI, kg/m^2^) and history of chronic diseases. BMI was determined as the specific value of weight (kg) to the square of height (m^2^) and categorized into four classifications depending on the standards suggested by the China Working Group on Obesity: underweight (<18.5), normal (18.5–23.9), overweight (24–27.9), and obese (≥28.0) [40]. Diagnoses of coronary heart disease (CHD), chronic obstructive pulmonary disease (COPD), asthma, and chronic bronchitis were determined by self-reporting. The definition of hypertension was measurements of systolic blood pressure ≥140 mmHg, measurements of diastolic blood pressure ≥ 90 mmHg, or prior diagnosis of hypertension [41]. The definition of diabetes was glycosylated hemoglobin type A1C (HbA1C) ≥ 6.5% (≥48 mmol/mol), fasting blood glucose (FPG) ≥ 126 mg/dL (≥7.0 mmol/L), or prior diagnosis of diabetes [42]. Definition of prevalent hyperlipidemia was measurements of triglyceride ≥ 1.70 mmol/L, measurements of total cholesterol ≥ 5.20 mmol/L, measurements of low-density lipoprotein cholesterol ≥ 3.40 mmol/L, measurements of high-density lipoprotein cholesterol < 1.00 mmol/L, or prior diagnosis of hyperlipidemia by a physician [17].

### 2.5. Statistical Analyses

Follow-up person-years of subjects enrolled were computed from the day of recruitment until the first observation of hyperuricemia in linked systems, or truncated at the earliest instance of death from other medical conditions or the termination of the study period (28 February 2023). The epidemiological metrics pertaining to hyperuricemia were delineated through the computation of the incidence density and cumulative incidence rate. The incidence density trends of hyperuricemia events were calculated by the nonparametric trend test.

Continual variables were identified by mean (standard deviation) or median (interquartile range), whereas category variables were identified by frequency (percentage). The correlations of baseline characteristics between the three DDS category groups were accessed by utilizing the one-way analysis of variance or the χ^2^ test depending on the circumstance.

We used the Cox proportional hazards models to examine the potential correlations between DDS and hyperuricemia occurrence, and to evaluate the hazard ratio (HR) and 95% confidence intervals (CIs) of hyperuricemia. The proportionate hazard was assessed utilizing the Schoenfeld individual test. In this regression model, days were used as the time scale, and the low-DDS group was considered as the reference group. Initially, model 1 was adjusted for sociodemographic factors comprising age, gender, educational attainment, marital situation, and retirement status. Then, model 2 was additionally adjusted for smoking, alcohol consumption, tea intake, PA, sleep time, dietary energy intake, and BMI. Next, model 3 was additionally adjusted for the histories of chronic diseases, such as diabetes, hypertension, CHD, COPD, dyslipidemia, chronic bronchitis, and asthma. Finally, employing the fully adjusted model (model 3), we addressed the median values across each DDS group as continual variables to examine the linear tendency, and re-analyzed the association of dietary diversity with hyperuricemia by eliminating participants who were discovered as having hyperuricemia within the first year or the initial two years of follow-up and by eliminating participants under 40 years old to validate the robustness of outcomes.

Subgroup analyses were undertaken to examine the potential correlation of dietary diversification with hyperuricemia differing by gender, age, smoking status, alcohol consumption, BMI, hypertension, CHD, diabetes, and dyslipidemia. Each subgroup assessment was adjusted for the whole covariates of the fully adjusted model except the covariate being analyzed. We also investigated the dose–response association between continuous variable DDS and hyperuricemia using restricted cubic splines (RCS) with all covariates fully adjusted.

Principles set forth by Strengthening the Reporting of Observational Studies in Epidemiology (STROBE) guidelines were followed in our research. All analyses were performed by the application of SPSS version 19.0 (SPSS Inc., Chicago, IL, USA) and R version 4.3.2 (R Development Core Team, Vienna, Austria). Two-sided statistical analyses were carried out, and a *p*-value lower than 0.05 implied a statistically significant disparity.

## 3. Results

### 3.1. Baseline Characteristics and Hyperuricemia Incidence

Table 1 portrays the characteristics at baseline of the 43,493 individuals participating in this study based on the three DDS group classifications. Overall, the study sample comprised 36.16% male participants, with the median age being 58 (50–65) years old. In contrast to participants in the low-DDS group, participants in the high-DDS group were more likely to be female and younger, have higher educational attainment and higher PA level, sleep for 5–8 h, and have a higher dietary energy intake. On the other hand, participants within the context of the high-DDS group were much less inclined to smoke or drink alcohol, and they also had a diminished prevalence rate of being overweight or obese or of having hypertension, diabetes, chronic bronchitis, or asthma.

**Figure 2:**
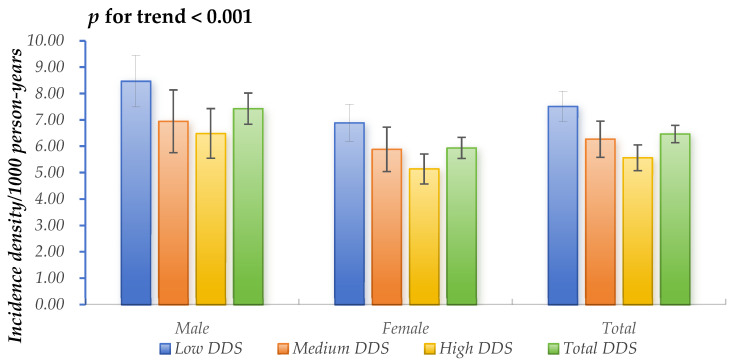
Incidence density of hyperuricemia across the three DDS category groups.

A total of 1460 newly developed hyperuricemia cases were ascertained throughout a median follow-up period of 5.59 years, encompassing 225,567.75 person-years as a whole. The overall incidence density was calculated at 6.47/1000 person-years (95% CIs: 6.14–6.80/1000 person-years), and the cumulative incidence rate was 3.4% (95% CIs: 3.2–3.5%). Individuals in the high-DDS group had the lowest incidence density of hyperuricemia, whereas those in the low-DDS group had the highest incidence density, as shown in Figure 2.

### 3.2. Association of Dietary Diversity Score with Hyperuricemia

The findings presented in Table 2 imply an inverse correlation between higher-DDS groups and the risk of developing hyperuricemia. In the non-adjusted model, the hazard ratios (HRs) for the medium- and high-DDS groups compared to the low-DDS group were 0.85 (95% CI 0.74–0.97) and 0.76 (95% CI 0.68–0.86), respectively. Having a medium DDS reduced the risk of incident hyperuricemia compared to the low DDS in model 2 and model 3 [HRs and 95% CIs were 0.87 (0.76–0.99)]. All models demonstrated that a high DDS had a beneficial effect in reducing the risk of incident hyperuricemia by 18%, 21%, and 20% compared to a low DDS. After considering multiple potential confounding factors in model 3, the risk of hyperuricemia incidence was reduced by 5% for each 1 unit of DDS increase. Comparable results were discovered in models 1 and 2. Trend analyses were statistically significant in all the models (*p* for trend < 0.001 or *p* for trend = 0.001).

The results sustained the consistency when individuals who received diagnoses of hyperuricemia within the first year and the first two years or participants aged 40 and below were eliminated after the baseline survey. In any of the aforementioned sensitivity analyses, the inverse correlation of DDS with the risk of developing hyperuricemia did not change fundamentally, with details provided in Supplementary Table S1.

### 3.3. Subgroup Analyses

**Figure 3:**
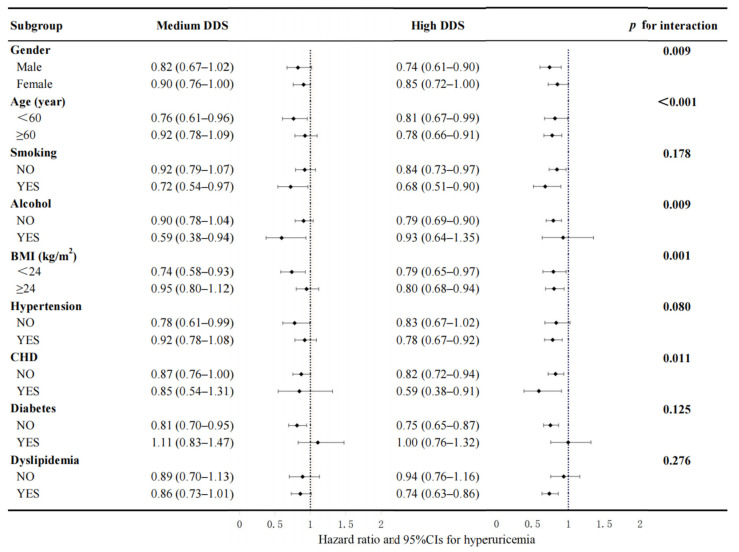
Association between the DDS group and hyperuricemia events by subgroups of gender, age, smoking status, BMI, alcohol consumption, diabetes, hypertension, dyslipidemia, and CHD. This model was calibrated for gender, age, educational attainment, marital situation, retirement status, smoking status, alcohol consumption, tea intake, PA, sleep time, BMI, dietary energy intakes, and the history of chronic diseases (including CHD, diabetes, hypertension, dyslipidemia, COPD, asthma, and chronic bronchitis). Every single subgroup analysis was adjusted for all the aforementioned covariates with the exception of the covariate being analyzed. Bold text is employed to represent the summary value of the subgroups.

Figure 3 reveals the correlation among the DDS groups and hyperuricemia incidence in each subgroup with all covariates fully adjusted except for the covariate being analyzed. No significant interplay was discovered between DDS and smoking, diabetes, hypertension, and hyperlipidemia. However, a significant interplay was discovered between the DDS group and hyperuricemia in the subgroups of age, gender, alcohol consumption, BMI, and CHD. The correlation between the high-DDS group and hyperuricemia seemed to be stronger among the participants who were male, over 60 years old, reported no alcohol consumption, had a BMI ≥ 24 kg/m^2^, and had CHD at baseline compared with their counterparts.

### 3.4. Dose–Response Analysis of Dietary Diversity Score with Hyperuricemia

**Figure 4:**
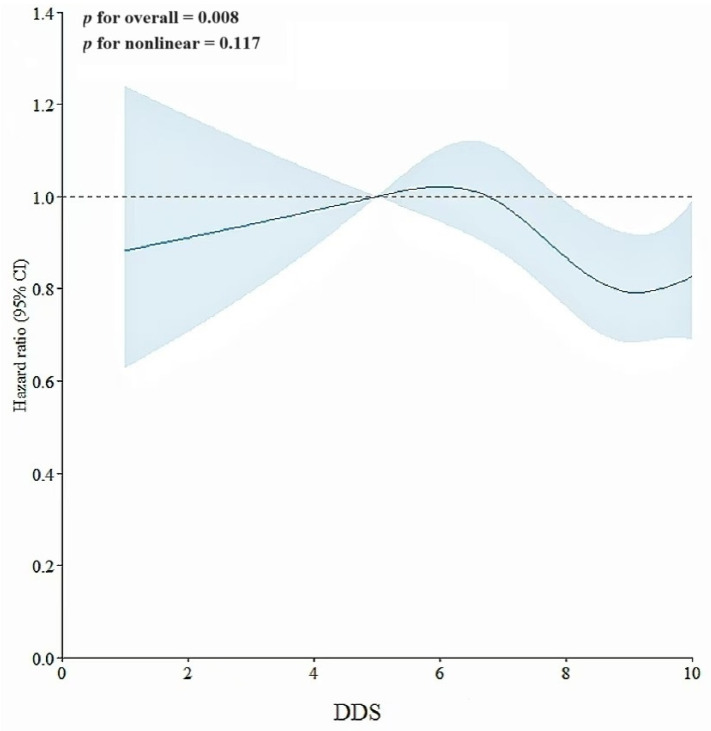
Association of DDS with hyperuricemia in RCS assessment. This model was adjusted for age, gender, educational attainment, marital situation, retirement status, smoking status, alcohol consumption, tea intake, PA, sleep time, BMI, dietary energy intake, and the history of chronic diseases (including diabetes, hypertension, CHD, COPD, dyslipidemia, asthma, and chronic bronchitis). Note: point estimates (blue line) and 95% CIs (light blue shaded area) were based on Cox regression models of the RCS with 4 knots at 5th, 35th, 65th, and 95th percentiles.

The RCS assessment revealed a linear correlation of DDS with hyperuricemia (*p* for linear tendency = 0.008, *p* for nonlinear tendency = 0.117; details are shown in Figure 4). The results remained consistent with the previous linear trend test. We further observed that an elevated DDS level was correlated with a decreased risk of hyperuricemia when DDS was above 7 points, which was consistent with the results achieved through Cox proportional hazards models.

## 4. Discussion

Our results illustrate an advantageous impact of higher dietary diversity on likelihood of hyperuricemia incidence among Chinese adults. In particular, there was a significant linear correlation between the consumption of more than seven food categories and the risk of hyperuricemia. As far as we are aware, this is the first attempt to offer convincing and substantial evidence for the correlation of dietary diversity with hyperuricemia in China. Our results further back the dietary concept of “food diversification” advised in the Dietary Guidelines for Chinese Residents (2022) [35].

The total incidence rate was 6.47/1000 person-years (95% CIs: 6.14–6.80/1000 person-years) in our study, which is consistent with our previous study [17]. Nevertheless, this result was far lower than the incidence rates, estimated to be 48.7–78/1000 person-years, obtained from additional cohort studies [43,44,45]. Causes of the differences were explained in our prior study and included participants being mostly women and challenges in recognizing individuals with asymptomatic hyperuricemia by application of regional systems for health information, etc. [17]. Furthermore, this study mainly focused on hyperuricemia without consideration of related diseases such as gout, which may cause a relative decreased incidence rate. This discrepancy suggests that hyperuricemia’s disease burden may be just a drop in the bucket. Although treatment is generally not warranted for asymptomatic hyperuricemia, it is important to emphasize that early recognition of asymptomatic hyperuricemia, however, provides an opportunity to modify or correct the underlying causes of hyperuricemia through diet and lifestyle given the differences in international guidelines, low diagnostic rates, and high levels of neglect [46].

In this study, we used DDS to indicate the dietary diversity. This indicator is mainly obtained by calculating the general number of food groups or the level of food intake, representing the total quality of diet and nutritional sufficiency compared with the assessment of specific foods or nutritive substance [47,48]. Although many indicators to capture dietary quality have been developed, including the Mediterranean diet [49], the DASH diet [50], and the dietary inflammatory index [51], these indicators tend to capture specific characteristics of diet rather than focusing on overall variation in diet [52]. Given the intricacy involved in measuring the abovementioned indices, the DDS appears to have more utility and be a more accessible approach that does not require quantitative measurements [27].

Our results emphasize the necessity of retaining a high DDS to decrease the risk of hyperuricemia. Limited empirical studies, as far as we are aware, have explored the correlation of dietary diversification with risk of hyperuricemia on the basis of a prospective cohort. Thus, the mechanism of negative associations of high DDS with hyperuricemia remains uncertain. A higher DDS, however, was indicated to have a positive impact on risk of chronic noncommunicable diseases, including abdominal obesity [47], hypertriglyceridemia [47], and type 2 diabetes [51,53]. Research showed that triglyceride, dyslipidemia, and abdominal obesity were associated with higher risks of hyperuricemia [47,54]. There was a mutually interdependent influence of hyperuricemia and type 2 diabetes on increased incidents as well as a reciprocal causative correlation between resistance to insulin and hyperuricemia [55,56]. The unclear causal relationship between hyperuricemia and other chronic diseases is expected to be elucidated in the future. Last but not least, a shift to a low dietary diversity predicts a decrease in the gut microbiota [36]. However, the gut microbiome, which facilitates the catabolism of purine and uric acid, was discovered to be a new target for treatment of hyperuricemia [57]. Therefore, dietary diversity is also an indispensable intervention factor associated with hyperuricemia prevention.

An interesting finding in the dose–response curve was that the intake of 8–10 broad categories of food could decrease the incidence of hyperuricemia. Nonetheless, the curve’s ultimate trend grew at a slow pace. Dietary diversification analysis may rarely consider the foods’ “healthfulness” within food groups [58]. Excessive overall dietary diversification not only increases the total amount of food intake, but may also increase the consumption of unhealthy foods [59]. Although the next turning point of the curve is unknown, we can cautiously predict that excessive dietary diversity may actually increase the risk of hyperuricemia. It is advocated to restrict the consumption of purines based on a moderate intake of multiple foods, which coincides with the guideline related to hyperuricemia in China [60]. A further suggestion proposed by the guidelines is that the daily food intake should include no fewer than 12 types per day and no fewer than 25 types per week. Ways should be practiced in daily life to achieve better food diversity, such as “small portion food consumption”, the “similar foods exchange based on foodstuff substitution method”, and “collocation of meat and vegetable” [35].

To our knowledge, this is the first study to provide convincing evidence for the correlation of dietary diversification with hyperuricemia among Chinese adults. This prospective cohort covered a very large, reliable sample that was closely linked with multiple health information systems to minimize follow-up losses. The large sample size allowed us to adjust confounding factors and ensured the robustness of test results through sensitivity analysis in various situations.

Our study has limitations as well. Firstly, the data used to calculate the DDS came from self-reporting, which might include recall bias. Nonetheless, to mitigate the bias, we also endeavored to take measures such as using a structured questionnaire conducted by trained professional technicians, restricting food categories in the FFQ based on eating habits in Shanghai, obtaining DDS based on easily recalled intake frequency rather than specific intakes, and excluding participants with abnormal energy intake. Moreover, the DDS was constructed based solely on whether the participants consumed the mentioned 10 food groups, without considering the quantity and “healthfulness” of foods, which might not accurately represent the true function of dietary variety. Priority will be given to future studies incorporating the level of dietary intake. Secondly, we did not consider the possible changes of participants’ dietary habits over time in this study due to the relatively short follow-up time (median 5.59 years). Time-dependent covariates such as shifting lifestyle characteristics (smoking cessation or weight changes) were difficult to capture and were not included in the study due to the fact that follow-up was connected through health information systems. It is currently unclear what role these covariates play in the correlation between dietary diversity and hyperuricemia. However, as the follow-up continues, additional dynamic data will be used for further research to fully consider these changes. Thirdly, the hyperuricemia incidence may have been underrated since we only included diagnosed cases of hyperuricemia and not the data from physical examinations; some asymptomatic patients might not seek medical attention. Fourthly, notwithstanding the number of data, the observational nature of the data precluded the opportunity to derive direct cause-and-effect risk. Nevertheless, this is our first exploration of the relationship between food diversity and hyperuricemia. Additional studies such as randomized controlled trials are expected to be undertaken in the future. Once sufficient studies have been completed, meta-analysis will be carried out to strengthen this evidence. Fifthly, the increasing use of over-the-counter (OTC) supplements like probiotics and cellulose was not taken into account in our study. Previous studies revealed that consuming probiotics or fiber could ameliorate hyperuricemia through modulating the gut microbiota or encouraging the excretion of uric acid [61,62,63]. We therefore venture to conclude that our results will not be significantly altered by the addition of OTC supplement into the diversified diet. Finally, this study was conducted among adults in Shanghai between the ages of 20 and 74, which might not be a complete representation of the whole Chinese population. Shanghai inhabitants reside on the east coast of China, have an obvious preference for animal food, dairy products, fruits, and vegetables, and consume more aquatic products than those in other regions [64]. Nevertheless, Shanghai, which has a sophisticated economy, is extensively urbanized and impacted by Western dietary practices [65]. In some ways, the shift in Shanghai inhabitants’ food pattern reflects a general trend of the evolution of Chinese citizens’ food pattern. It demonstrates the significance of encouraging dietary diversity nationwide to prevent hyperuricemia. Given the large sample size, our study can serve as a cohort reference for comparative dietary analysis. Even so, additional caution is warranted when extending these findings to different demographics or nations.

## 5. Conclusions

In summary, a higher DDS (especially above seven categories) reduced the risk of incident hyperuricemia compared to a lower DDS among Chinese inhabitants residing in Shanghai. Results support the dietary principle of “food diversification” suggested by guidelines. Our conclusions should be applied with caution given that few studies have assessed the relationship between DDS and hyperuricemia. We call for further research incorporating food-specific information and a longer follow-up period to provide additional evidence.

## Figures and Tables

**Table 1:** Baseline characteristics of participants according to the three DDS category groups.

Characteristics	Total	Low DDS (0–7)	Medium DDS (8)	High DDS (9–10)	*p*-Value
	(*n* = 43,493)	(*n* = 16,014)	(*n* = 9805)	(*n* = 17,674)	
Newly developed hyperuricemia (%)	1460 (3.36)	647 (4.04)	320 (3.26)	493 (2.79)	<0.001
Male (%)	15,727 (36.16)	6361 (39.72)	3666 (37.39)	5700 (32.25)	<0.001
Age (year)	58 (50–65)	59 (52–65)	58 (50–65)	57 (48–64)	<0.001
Age (group)					<0.001
20–39	4892 (11.25)	1343 (8.39)	1096 (11.18)	2453 (13.88)	
40–49	5556 (12.77)	1812 (11.32)	1290 (13.16)	2454 (13.88)	
50–59	13,438 (30.90)	5078 (31.71)	3060 (31.21)	5300 (29.99)	
60–69	15,557 (35.77)	6158 (38.45)	3502 (35.71)	5897 (33.37)	
70–74	4050 (9.31)	1623 (10.13)	857 (8.74)	1570 (8.88)	
Educational attainment (%)					<0.001
Primary school or below	13,810 (31.75)	6884 (42.99)	3135 (31.97)	3791 (21.45)	
Junior high school	16,991 (39.07)	5944 (37.12)	3962 (40.41)	7085 (40.09)	
Senior high school or above	12,692 (29.18)	3186 (19.89)	2708 (27.62)	6798 (38.46)	
Marriage situation (%)					
Unmarried	830 (1.91)	273 (1.70)	193 (1.97)	364 (2.06)	<0.001
Married	39,867 (91.66)	14,579 (91.04)	8952 (91.3)	16,336 (92.43)	
Divorced and other	2796 (6.43)	1162 (7.26)	660 (6.73)	974 (5.51)	
Retirement (%)	26,547 (61.04)	10,004 (62.47)	5881 (59.98)	10,662 (60.33)	<0.001
Smoking (%)	8656 (19.90)	3968 (24.78)	1999 (20.39)	2689 (15.21)	<0.001
Alcohol consumption (%)	4525 (10.40)	2000 (12.49)	1072 (10.93)	1453 (8.22)	<0.001
Tea intake (%)	12,845 (29.53)	4596 (28.70)	2994 (30.54)	5255 (29.73)	0.005
PA level (%)					<0.001
Low	24,803 (57.03)	10,504 (65.59)	5611 (57.23)	8688 (49.16)	
Moderate	14,496 (33.33)	4354 (27.19)	3276 (33.41)	6866 (38.85)	
High	4194 (9.64)	1156 (7.22)	918 (9.36)	2120 (11.99)	
Sleeping time (%)					<0.001
<5 h	2067 (4.75)	931 (5.82)	434 (4.43)	702 (3.97)	
5–8 h	33,501 (77.03)	11,910 (74.37)	7554 (77.04)	14,037 (79.42)	
≥8 h	7925(18.22)	3173 (19.81)	1817 (18.53)	2935 (16.61)	
BMI (kg/m^2^)	23.95 ± 3.25	24.09 ± 3.29	23.98 ± 3.21	23.82 ± 3.22	<0.001
BMI (%)					<0.001
Underweight	1453 (3.34)	515 (3.22)	324 (3.31)	614 (3.47)	
Normal Weight	21,577(49.61)	7672 (47.91)	4842 (49.38)	9063 (51.28)	
Overweight	15,948 (36.67)	6023 (37.61)	3623 (36.95)	6302 (35.66)	
Obese	4515 (10.38)	1804 (11.26)	1016 (10.36)	1695 (9.59)	
Energy intake (kcal/d)	1142.79 (909.34–1482.55)	1008.75 (830.18–1292.32)	1133.62 (912.03–1450.63)	1280.47 (1022.72–1634.33)	<0.001
History of chronic diseases (%)					
Hypertension	20,715 (47.63)	8053 (50.29)	4708 (48.02)	7954 (45.00)	<0.001
CHD	1972 (4.53)	699 (4.36)	468 (4.77)	805 (4.55)	0.306
Diabetes	6431 (14.79)	2587 (16.15)	1454 (14.83)	2390 (13.52)	<0.001
Dyslipidemia	24,835 (57.10)	9113 (56.91)	5613 (57.25)	10,109 (57.20)	0.819
COPD	241 (0.55)	87 (0.54)	54 (0.55)	100 (0.57)	0.961
Chronic bronchitis	2969 (6.83)	1240 (7.74)	663 (6.76)	1066 (6.03)	<0.001
Asthma	1033 (2.38)	416 (2.60)	250 (2.55)	367 (2.08)	0.003

The mean (standard deviation) is presented for continual variables, while the frequency (percentage) is presented for category variables. The analysis of variance or non-parametric Kruskal–Wallis H test was utilized for continual variables, and the Mantel–Haenszel χ^2^ test was utilized for category variables. PA, physical activity; CHD, coronary heart disease; BMI, body mass index; COPD, chronic obstructive pulmonary disease.

**Table 2:** HRs (95% CIs) of hyperuricemia by the three DDS category groups.

	Per Unit Increase in DDS	DDS Group			*p* for Trend
		Low (0–7)	Medium (8)	High (9–10)	
Non-Adjusted Model	0.94 (0.91–0.97) *	1.00	0.85 (0.74–0.97) *	0.76 (0.68–0.86) *	<0.001
Adjusted Model 1	0.96 (0.93–0.99) *	1.00	0.88 (0.77–1.01)	0.82 (0.72–0.92) *	0.001
Adjusted Model 2	0.95 (0.92–0.99) *	1.00	0.87 (0.76–0.99) *	0.79 (0.70–0.90) *	<0.001
Adjusted Model 3	0.95 (0.92–0.99) *	1.00	0.87 (0.76–0.99) *	0.80 (0.70–0.91) *	<0.001

The median value in every single category was considered as a continual variable to compute linear tendency. Model 1: adjusted for sociological demographic characteristics including age, gender, educational attainment, marital situation, and retirement status. Model 2: additionally adjusted for smoking status, alcohol consumption, tea intake, PA, sleep time, dietary energy intake, and BMI. Model 3: additionally adjusted for the histories of chronic diseases, such as hypertension, diabetes, CHD, COPD, dyslipidemia, chronic bronchitis, and asthma. The categorization of DDS was informed by its practical public health implications. * *p* < 0.05.

## Data Availability

All data that support the findings of this study are included in this manuscript and its Supplementary Materials files.

## Supplementary Materials

The following supporting information can be downloaded at https://www.mdpi.com/article/10.3390/nu16172968/s1: Figure S1: Association of DDS with hyperuricemia in restricted cubic spline models. The model was adjusted for no covariates; Table S1: The association (HRs, 95% CIs) between DDS groups with incidence of hyperuricemia the first year and the first two years after baseline survey or after excluding participants aged under 40 at the baseline survey.

## Abbreviations

DASH	Dietary approaches to stop hypertension
DDS	Dietary Diversity Score
SSACB	Shanghai Suburban Adult Cohort and Biobank
EMR	Electronic Medical Record System
CDM	Chronic Disease Management System
CR	Cancer Registry System
CDSS	Cause-of-Death Surveillance System
FFQ	Food frequency questionnaire
ICD-10	International Classification of Diseases tenth revision
PA	Physical activity
IPAQ	International Physical Activity Questionnaire
BMI	Body mass index
CHD	Coronary heart disease
COPD	Chronic obstructive pulmonary disease
HbA1C	Hemoglobin type A1C
FPG	Fasting blood glucose
HR	Hazard ratio
95% CIs	95% confidence intervals
RCS	Restricted cubic splines
STROBE	Strengthening the Reporting of Observational Studies in Epidemiology
OTC	Over the counter

## References

[1] Dalbeth N., Gosling A.L., Gafo A., Abhishek A. (2021). Gout. Lancet.

[2] Mortada I. (2017). Hyperuricemia, type 2 diabetes mellitus, and hypertension: An emerging association. Curr. Hypertens. Rep..

[3] Kim J.H., Kwon M.J., Choi H.G., Lee S.J., Kim S.W., Kim J.H., Kwon B.C., Lee J.W. (2022). The association between hyperuricemia and cardiovascular disease history: A cross-sectional study using KoGES HEXA data. Medicine.

[4] Prasad Sah O.S., Qing Y.X. (2015). Associations between hyperuricemia and chronic kidney disease: A review. Nephrourol Mon..

[5] Kikuchi A., Kawamoto R., Ninomiya D., Kumagi T. (2022). Hyperuricemia is associated with all-cause mortality among males and females: Findings from a study on Japanese community-dwelling individuals. Metabol. Open.

[6] Otaki Y., Konta T., Ichikawa K., Fujimoto S., Iseki K., Moriyama T., Yamagata K., Tsuruya K., Narita I., Kondo M. (2021). Possible burden of hyperuricaemia on mortality in a community-based population: A large-scale cohort study. Sci. Rep..

[7] Yokose C., McCormick N., Choi H.K. (2021). The role of diet in hyperuricemia and gout. Curr. Opin. Rheumatol..

[8] Chen-Xu M., Yokose C., Rai S.K., Pillinger M.H., Choi H.K. (2019). Contemporary Prevalence of Gout and Hyperuricemia in the United States and Decadal Trends: The National Health and Nutrition Examination Survey, 2007–2016. Arthritis Rheumatol..

[9] Kumar A.U.A., Browne L.D., Li X., Adeeb F., Perez-Ruiz F., Fraser A.D., Stack A.G. (2018). Temporal trends in hyperuricaemia in the Irish health system from 2006-2014: A cohort study. PLoS ONE.

[10] Pathmanathan K., Robinson P.C., Hill C.L., Keen H.I. (2021). The prevalence of gout and hyperuricaemia in Australia: An updated systematic review. Semin. Arthritis Rheum..

[11] Zhang M., Zhu X., Wu J., Huang Z., Zhao Z., Zhang X., Xue Y., Wan W., Li C., Zhang W. (2022). Prevalence of Hyperuricemia Among Chinese Adults: Findings from Two Nationally Representative Cross-Sectional Surveys in 2015-16 and 2018-19. Front. Immunol..

[12] Li S., Liu X., Jia X., Fang M., Yang Q., Gong Z. (2023). Assessment of the temporal trend and daily profiles of the dietary purine intake among Chinese residents during 2014 to 2021. Front. Nutr..

[13] Kuwabara M., Fukuuchi T., Aoki Y., Mizuta E., Ouchi M., Kurajoh M., Maruhashi T., Tanaka A., Morikawa N., Nishimiya K. (2023). Exploring the Multifaceted Nexus of Uric Acid and Health: A Review of Recent Studies on Diverse Diseases. Biomolecules.

[14] Li R., Yu K., Li C. (2018). Dietary factors and risk of gout and hyperuricemia: A meta-analysis and systematic review. Asia Pac. J. Clin. Nutr..

[15] Gao Y., Cui L.F., Sun Y.Y., Yang W.H., Wang J.R., Wu S.L., Gao X. (2021). Adherence to the dietary approaches to stop hypertension diet and hyperuricemia: A cross-sectional study. Arthritis Care Res..

[16] Yokose C., McCormick N., Choi H.K. (2021). Dietary and Lifestyle-Centered Approach in Gout Care and Prevention. Curr. Rheumatol. Rep..

[17] Yi K., Cui S., Tang M., Wu Y., Xiang Y., Yu Y., Tong X., Jiang Y., Zhao Q., Zhao G. (2022). Adherence to DASH Dietary Pattern and Its Association with Incident Hyperuricemia Risk: A Prospective Study in Chinese Community Residents. Nutrients.

[18] Phillips J.A. (2021). Dietary Guidelines for Americans, 2020–2025. Workplace Health Saf..

[19] Springmann M., Spajic L., Clark M.A., Poore J., Herforth A., Webb P., Rayner M., Scarborough P. (2020). The healthiness and sustainability of national and global food based dietary guidelines: Modelling study. BMJ.

[20] O’Hearn M., Erndt-Marino J., Gerber S., Lauren B.N., Economos C., Wong J.B., Blumberg J.B., Mozaffarian D. (2022). Validation of Food Compass with a healthy diet, cardiometabolic health, and mortality among U.S. adults, 1999–2018. Nat. Commun..

[21] Gardner C.D., Vadiveloo M.K., Petersen K.S., Anderson C.A.M., Springfield S., Van Horn L., Khera A., Lamendola C., Mayo S.M., Joseph J.J. (2023). Popular Dietary Patterns: Alignment With American Heart Association 2021 Dietary Guidance: A Scientific Statement from the American Heart Association. Circulation.

[22] Mente A., Dehghan M., Rangarajan S., O’Donnell M., Hu W., Dagenais G., Wielgosz A., A Lear S., Wei L., Diaz R. (2023). Diet, cardiovascular disease, and mortality in 80 countries. Eur. Heart J..

[23] Kennedy G., Ballard T., Dop M.-C. (2011). Guidelines for Measuring Household and Individual Dietary Diversity.

[24] Foote J.A., Murphy S.P., Wilkens L.R., Basiotis P.P., Carlson A. (2004). Dietary variety increases the probability of nutrient adequacy among adults. J. Nutr..

[25] de Oliveira Otto M.C., Anderson C.A.M., Dearborn J.L., Ferranti E.P., Mozaffarian D., Rao G., Wylie-Rosett J., Lichtenstein A.H., American Heart Association Behavioral Change for Improving Health Factors Committee of the Council on Lifestyle and Cardiometabolic Health and Council on Epidemiology and Prevention, Council on Cardiovascular and Stroke Nursing (2018). Dietary Diversity: Implications for Obesity Prevention in Adult Populations: A Science Advisory from the American Heart Association. Circulation.

[26] Zheng G., Xia H., Shi H., Zheng D., Wang X., Ai B., Tian F., Lin H. (2024). Effect modification of dietary diversity on the association of air pollution with incidence, complications, and mortality of type 2 diabetes: Results from a large prospective cohort study. Sci. Total Environ..

[27] Zhong W.F., Song W.Q., Wang X.M., Li Z.H., Shen D., Liu D., Zhang P.D., Shen Q.Q., Liang F., Nan Y. (2023). Dietary Diversity Changes and Cognitive Frailty in Chinese Older Adults: A Prospective Community-Based Cohort Study. Nutrients.

[28] Wang X.M., Zhong W.F., Li Z.H., Chen P.L., Zhang Y.J., Ren J.J., Liu D., Shen Q.Q., Yang P., Song W.Q. (2023). Dietary diversity and frailty among older Chinese people: Evidence from the Chinese Longitudinal Healthy Longevity Study. Am. J. Clin. Nutr..

[29] Lv Y., Kraus V.B., Gao X., Yin Z., Zhou J., Mao C., Duan J., Zeng Y., Brasher M.S., Shi W. (2020). Higher dietary diversity scores and protein-rich food consumption were associated with lower risk of all-cause mortality in the oldest old. Clin. Nutr..

[30] Torres-Collado L., García-de la Hera M., Cano-Ibañez N., Bueno-Cavanillas A., Vioque J. (2022). Association between Dietary Diversity and All-Cause Mortality: A Multivariable Model in a Mediterranean Population with 18 Years of Follow-Up. Nutrients.

[31] Zhao Q., Chen B., Wang R., Zhu M., Shao Y., Wang N., Liu X., Zhang T., Jiang F., Wang W. (2020). Cohort profile: Protocol and baseline survey for the Shanghai Suburban Adult Cohort and Biobank (SSACB) study. BMJ Open.

[32] Shan Z., Li Y., Baden M.Y., Bhupathiraju S.N., Wang D.D., Sun Q., Rexrode K.M., Rimm E.B., Qi L., Willett W.C. (2020). Association Between Healthy Eating Patterns and Risk of Cardiovascular Disease. JAMA Intern. Med..

[33] Zhu Y., An Q., Rao J. (2024). The effects of dietary diversity on health status among the older adults: An empirical study from China. BMC Public Health.

[34] Duan Y., Qi Q., Cui Y., Yang L., Zhang M., Liu H. (2023). Effects of dietary diversity on frailty in Chinese older adults: A 3-year cohort study. BMC Geriatr..

[35] Chinese Nutrition Society (2022). Dietary Guidelines for Chinese Residents (2022).

[36] Zhang J., Liang D., Zhao A. (2020). Dietary Diversity and the Risk of Fracture in Adults: A Prospective Study. Nutrients.

[37] Borghi C., Domienik-Karłowicz J., Tykarski A., Widecka K., Filipiak K.J., Jaguszewski M.J., Narkiewicz K., Mancia G. (2021). Expert consensus for the diagnosis and treatment of patient with hyperuricemia and high cardiovascular risk: 2021 update. Cardiol. J..

[38] Yang Y.X., Wang G.Y., Pan X.C. (2009). China Food Composition Table.

[39] Bassett D.R. (2003). International physical activity questionnaire: 12-country reliability and validity. Med. Sci. Sports Exerc..

[40] Yang Y., Zhang H., Lan X., Qin X., Huang Y., Wang J., Luo P., Wen Z., Li Y., Kong Y. (2022). Low BMI and high waist-to-hip ratio are associated with mortality risk among hemodialysis patients: A multicenter prospective cohort study. Clin. Kidney J..

[41] NCD Risk Factor Collaboration (NCD-RisC) (2021). Worldwide trends in hypertension prevalence and progress in treatment and control from 1990 to 2019: A pooled analysis of 1201 population-representative studies with 104 million participants. Lancet.

[42] American Diabetes Association Professional Practice Committee (2024). 2. Diagnosis and Classification of Diabetes: Standards of Care in Diabetes-2024. Diabetes Care.

[43] Ryu S., Chang Y., Zhang Y., Kim S.G., Cho J., Son H.J., Shin H., Guallar E. (2012). A cohort study of hyperuricemia in middle-aged South Korean men. Am. J. Epidemiol..

[44] Liang H., Zhang J., Yu H., Ding L., Liu F., Wang J. (2023). Incidence density of hyperuricemia and association between metabolism-related predisposing risk factors and serum urate in Chinese adults: A cohort study. Front. Endocrinol..

[45] Ni Q., Lu X., Chen C., Du H., Zhang R. (2019). Risk factors for the development of hyperuricemia: A STROBE-compliant cross-sectional and longitudinal study. Medicine.

[46] Li Q., Li X., Wang J., Liu H., Kwong J.S., Chen H., Li L., Chung S.C., Shah A., Chen Y. (2019). Diagnosis and treatment for hyperuricemia and gout: A systematic review of clinical practice guidelines and consensus statements. BMJ Open.

[47] Kim J., Kim M., Shin Y., Cho J.H., Lee D., Kim Y. (2022). Association between Dietary Diversity Score and Metabolic Syndrome in Korean Adults: A Community-Based Prospective Cohort Study. Nutrients.

[48] Karimbeiki R., Pourmasoumi M., Feizi A., Abbasi B., Hadi A., Rafie N., Safavi S.M. (2018). Higher dietary diversity score is associated with obesity: A case-control study. Public Health.

[49] Delgado-Lista J., Alcala-Diaz J.F., Torres-Peña J.D., Quintana-Navarro G.M., Fuentes F., Garcia-Rios A., Ortiz-Morales A.M., Gonzalez-Requero A.I., Perez-Caballero A.I., Yubero-Serrano E.M. (2022). Long-term secondary prevention of cardiovascular disease with a Mediterranean diet and a low-fat diet (CORDIOPREV): A randomised controlled trial. Lancet.

[50] Filippou C.D., Tsioufis C.P., Thomopoulos C.G., Mihas C.C., Dimitriadis K.S., Sotiropoulou L.I., Chrysochoou C.A., Nihoyannopoulos P.I., Tousoulis D.M. (2020). Dietary Approaches to Stop Hypertension (DASH) Diet and Blood Pressure Reduction in Adults with and without Hypertension: A Systematic Review and Meta-Analysis of Randomized Controlled Trials. Adv. Nutr..

[51] Zheng G., Cai M., Liu H., Li R., Qian Z., Howard S.W., Keith A.E., Zhang S., Wang X., Zhang J. (2023). Dietary Diversity and Inflammatory Diet Associated with All-Cause Mortality and Incidence and Mortality of Type 2 Diabetes: Two Prospective Cohort Studies. Nutrients.

[52] Moraeus L., Lindroos A.K., Warensjö Lemming E., Mattisson I. (2020). Diet diversity score and healthy eating index in relation to diet quality and socio-demographic factors: Results from a cross-sectional national dietary survey of Swedish adolescents. Public Health Nutr..

[53] Conklin A.I., Monsivais P., Khaw K.T., Wareham N.J., Forouhi N.G. (2016). Dietary Diversity, Diet Cost, and Incidence of Type 2 Diabetes in the United Kingdom: A Prospective Cohort Study. PLoS Med..

[54] Xu Y., Dong H., Zhang B., Zhang J., Ma Q., Sun H. (2022). Association between dyslipidaemia and the risk of hyperuricaemia: A six-year longitudinal cohort study of elderly individuals in China. Ann. Med..

[55] Lai H.M., Chen C.J., Su B.Y., Chen Y.C., Yu S.F., Yen J.H., Hsieh M.C., Cheng T.T., Chang S.J. (2012). Gout and type 2 diabetes have a mutual inter-dependent effect on genetic risk factors and higher incidences. Rheumatology.

[56] Li C., Hsieh M.C., Chang S.J. (2013). Metabolic syndrome, diabetes, and hyperuricemia. Curr. Opin. Rheumatol..

[57] Wang J., Chen Y., Zhong H., Chen F., Regenstein J., Hu X., Cai L., Feng F. (2022). The gut microbiota as a target to control hyperuricemia pathogenesis: Potential mechanisms and therapeutic strategies. Crit. Rev. Food Sci. Nutr..

[58] Du Q., Lu Y., Hu F., Feng X., Zhang Y., Li S., Zhang C., Zhang H., Zeng Y., Yao Y. (2023). Dietary diversity and possible sarcopenia among older people in China: A nationwide population-based study. Front. Nutr..

[59] Oliveira Otto M.C.D., Padhye N.S., Bertoni A.G., Jacobs D.R., Mozaffarian D. (2015). Everything in Moderation—Dietary Diversity and Quality, Central Obesity and Risk of Diabetes. PLoS ONE.

[60] National Health Commission of the People’s Republic of China, China National Center for Food Safety Risk Assessment (2024). Dietary guidelines for adult Hyperuricemia and gout (2024). J. Hyg. Res..

[61] Sun Y., Sun J., Zhang P., Zhong F., Cai J., Ma A. (2019). Association of dietary fiber intake with hyperuricemia in U.S. adults. Food Funct..

[62] Guo Y., Yu Y., Li H., Ding X., Li X., Jing X., Chen J., Liu G., Lin Y., Jiang C. (2021). Inulin supplementation ameliorates hyperuricemia and modulates gut microbiota in Uox-knockout mice. Eur. J. Nutr..

[63] Zhao H., Lu Z., Lu Y. (2022). The potential of probiotics in the amelioration of hyperuricemia. Food Funct..

[64] Lian Y., Gu L., Yang L., Wang L., Li H. (2023). The Reasonableness and Spatial Differences of the Food Consumption Structure of Urban and Rural Residents in China, 2015–2021. Foods.

[65] Howard A.G., Attard S.M., Herring A.H., Wang H., Du S., Gordon-Larsen P. (2021). Socioeconomic gradients in the Westernization of diet in China over 20 years. SSM Popul. Health.

