# Supplementary material for: The Association of Dietary Diversity with Hyperuricemia among Community Inhabitants in Shanghai, China: A Prospective Research
Source: Nutrients. 2024 Sep 3;16(17):2968. doi: 10.3390/nu16172968 (PMC11397405; doi:10.3390/nu16172968)
Supplement: Supplementary file 1 [file nutrients-16-02968-s001.zip › nutrients-3165692-supplementary.pdf]

Supplementary Materials

Article

The Association of Dietary Diversity with Hyperuricemia among Community Inhabitants in Shanghai, China: A Prospective Research

Table S1. The association (HRs, 95% CIs) between DDS groups with incident hyperuricemia the first year and the first two years after baseline survey, or after excluding participants aged under 40 at the baseline survey.

**Models**		**Per 1-unit Increase in DDS**	**DDS Group**			***p* for Trend**
			**Low (0-7)**	**Medium (8)**	**High (9-10)**	
Model4	Non-adjusted	0.93 (0.91-0.96)*	1.00	0.83 (0.72-0.95)*	0.75 (0.66-0.85)*	<0.001
	Adjusted Model 1	0.96 (0.93-0.99)*	1.00	0.87 (0.76-1.00)	0.81 (0.72-0.92)*	0.001
	Adjusted Model 2	0.95 (0.92-0.98)*	1.00	0.86 (0.75-0.99)*	0.80 (0.70-0.91)*	<0.001
	Adjusted Model 3	0.95 (0.92- 0.98)*	1.00	0.86 (0.74-0.99)*	0.80 (0.70-0.91)*	0.001
Model5	Non-adjusted	0.93 (0.90-0.96)*	1.00	0.80 (0.68-0.93)*	0.72 (0.63-0.83)*	<0.001
	Adjusted Model 1	0.95 (0.92-0.99)*	1.00	0.84 (0.72-0.98)*	0.80 (0.70-0.92)*	0.001
	Adjusted Model 2	0.95 (0.92, 0.99)*	1.00	0.83 (0.71-0.98)*	0.79 (0.69-0.92)*	0.001
	Adjusted Model 3	0.95 (0.92, 0.99)*	1.00	0.83 (0.71-0.98)*	0.80 (0.69-0.92)*	0.001

Models		Per 1-unit Increase in DDS	DDS Group			p for Trend
			Low (0-7)	Medium (8)	High (9-10)	

Table S1. Cont.

Models		Per 1-unit Increase in DDS	DDS Group			*p* for Trend
			Low (0-7)	Medium (8)	High (9-10)	
Model6	Non-adjusted	0.95 (0.92-0.98)*	1.00	0.87 (0.76-0.99)	0.80 (0.71-0.90)	<0.001
	Adjusted Model 1	0.96 (0.93-0.99)*	1.00	0.89 (0.77-1.02)	0.82 (0.72-0.93)	0.001
	Adjusted Model 2	0.95 (0.92-0.99)*	1.00	0.87 (0.76-1.00)	0.80 (0.70-0.91)	<0.001
	Adjusted Model 3	0.95 (0.92-0.99)*	1.00	0.87 (0.77-1.00)	0.80 (0.70-0.91)	0.001

The median value in every single category was considered as a continual variable to compute linear tendency. Model 1 : adjusted for sociological demographic characteristics including age, gender, educational attainment, marital situation, and retirement. Model 2: additionally adjusted for smoking, alcohol consumption, tea intake, PA, sleep time, dietary energy intake and BMI . Model 3: additionally adjusted the histories of chronic diseases, such as hypertension, diabetes, CHD, COPD, dyslipide-mia, chronic bronchitis, and asthma. The categorization of DDS was informed by its practical public health implications. *$p < 0.05$.

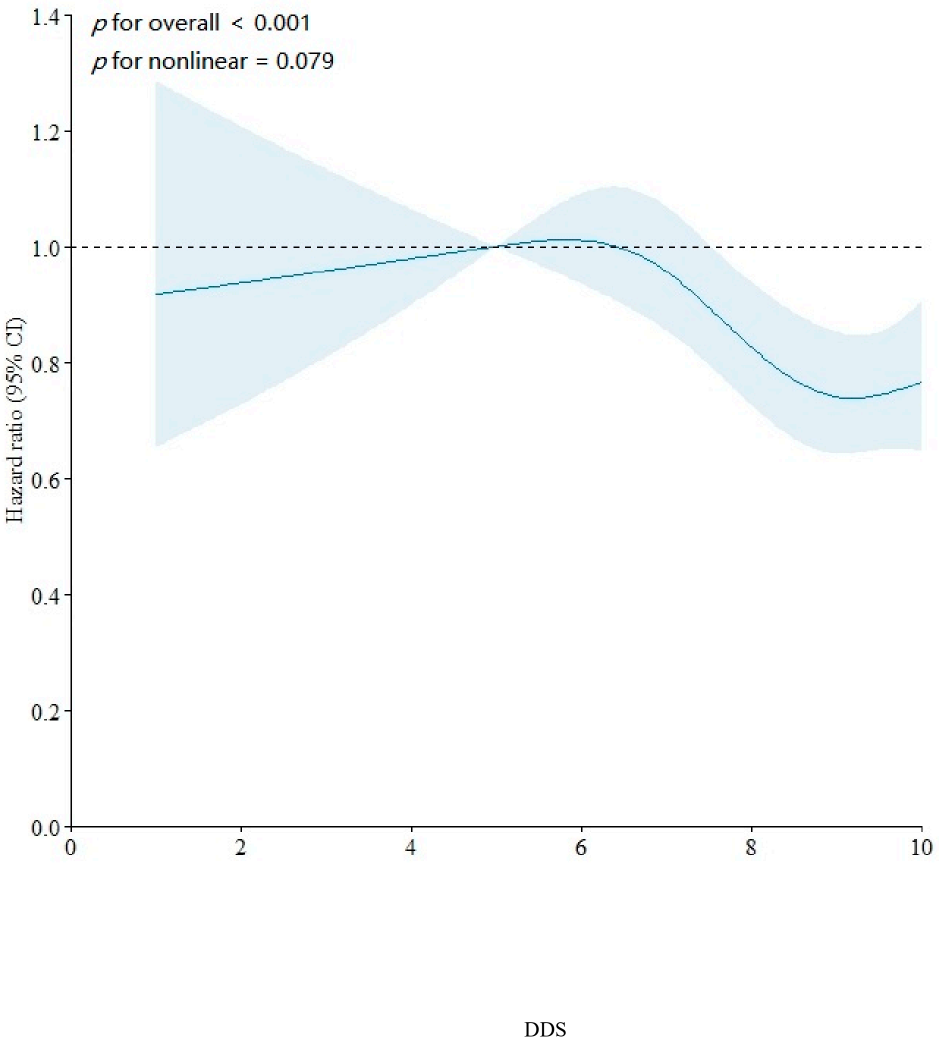

Figure S1. Association of DDS with hyperuricemia in restricted cubic spline models. The model was adjusted for no covariates. Point estimates (blue line) and 95%CI (light blue shaded area) were based on Cox regression models of the RCS with 4 knots at 5th, 35th, 65th, and 95th percentiles.
